# Altered radiation responses of breast cancer cells resistant to hormonal therapy

**DOI:** 10.18632/oncotarget.3188

**Published:** 2014-12-02

**Authors:** Lidiya Luzhna, Anne E. Lykkesfeldt, Olga Kovalchuk

**Affiliations:** ^1^ Department of Biological Sciences, University of Lethbridge, University Drive, Lethbridge, AB, Canada; ^2^ Breast Cancer Group, Cell Death and Metabolism, Danish Cancer Society Research Center, Strandboulevarden, Copenhagen, Denmark

**Keywords:** breast cancer, endocrine therapy, radiation, transcriptomics, apoptosis, treatment resistance

## Abstract

Endocrine therapy agents (the selective estrogen receptor (ER) modulators such as tamoxifen or the selective ER down-regulators such as ICI 182,780) are key treatment regimens for hormone receptor-positive breast cancers. While these drugs are very effective in controlling ER-positive breast cancer, many tumors that initially respond well to treatment often acquire drug resistance, which is a major clinical problem. In clinical practice, hormonal therapy agents are commonly used in combination or sequence with radiation therapy. Tamoxifen treatment and radiotherapy improve both local tumor control and patient survival. However, tamoxifen treatment may render cancer cells less responsive to radiation therapy.

Only a handful of data exist on the effects of radiation on cells resistant to hormonal therapy agents. These scarce data show that cells that were resistant to tamoxifen were also resistant to radiation. Yet, the existence and mechanisms of cross-resistance to endocrine therapy and radiation therapy need to be established.

Here, we for the first time examined and compared radiation responses of MCF-7 breast adenocarcinoma cells (MCF-7/S0.5) and two antiestrogen resistant cell lines derived from MCF-7/S0.5: the tamoxifen resistant MCF-7/TAM^R^-1 and ICI 182,780 resistant MCF-7/182^R^-6 cell lines. Specifically, we analyzed the radiation-induced changes in the expression of genes involved in DNA damage, apoptosis, and cell cycle regulation. We found that the tamoxifen-resistant cell line in contrast to the parental and ICI 182,780-resistant cell lines displayed a significantly less radiation-induced decrease in the expression of genes involved in DNA repair. Furthermore, we show that MCF-7/TAM^R^-1 and MCF-7/182^R^-6 cells were less susceptible to radiation-induced apoptosis as compared to the parental line. These data indicate that tamoxifen-resistant breast cancer cells have a reduced sensitivity to radiation treatment. The current study may therefore serve as a roadmap to the future analysis of the mechanisms of cross-resistance between hormonal therapy and radiation.

## INTRODUCTION

Endocrine therapy is a widely accepted treatment of choice for hormone receptor-positive breast cancers in early stages and during advanced metastasis [1]. Women with estrogen receptor- (ER) and/or progesterone receptor (PR) positive breast cancers are the best candidates for hormone therapy [2]. The ERα-positive normal breast cells may produce growth factors that stimulate the proliferation of neighboring cells leading to breast cancer development. In contrast, ERβ is essential for breast tissue differentiation, and its loss is associated with breast carcinogenesis [3]. The selective estrogen receptor modulators (SERMs) such as Tamoxifen bind to the ligand-binding domain (LBD) of ER preventing its stimulation by estrogen, while the selective estrogen receptor down-regulators (SERDs) such as ICI 182,780 (Fulvestrant, Faslodex) bind, block and increase the degradation of ER [3, 4]. Both drugs are currently established as effective treatment therapy with beneficial outcomes. Unfortunately, in the case of advanced disease, acquired resistance to both drugs inevitably develops, which is a major clinical problem [5-8]. Drug resistance is usually accompanied with an aggressive cell behavior and invasiveness. The evidence exists that the main mechanism of hormone therapy resistance is the deregulation of growth factor-signaling cascades. The over-expression of growth factors, their receptors and downstream signaling elements promotes hormone therapy failure [8-10]. Long-term estrogen-deprived tumor cells may adapt to low levels of estrogen by increasing their sensitivity to it [11]. Such enhanced sensitivity to estrogen may result from the activation of several signaling pathways such as RAS, RAF, MEK and MAPK [12, 13]. Moreover, it has been shown that tamoxifen- and fulvestrant- resistant MCF-7 cells overexpress receptors in the HER family, e.g. EGFR and HER2 [5-7, 9, 10, 14]. The overexpressed EGFR and HER2 are well known to recruit MAPK, AKT and PKC signaling cascades [15-17].

The combination of hormone therapy and radiation is widely used in clinical practice. The application of tamoxifen and radiotherapy is believed to improve both local control and patient survival [18, 19]. Nevertheless, a suspicion also exists that tamoxifen may render cancer cells less responsive to radiotherapy by providing a protective effect against radiation. Early studies on cell culture have shown that tamoxifen causes an arrest of cells in the radioresistant G0/G1 phase of the cell cycle reducing the radiosensitivity of tumor cells pretreated with tamoxifen [20-23]. Today, the most important clinical concern is the optimal scheduling (either concurrent or sequential) of radiation and hormonal therapy administration [24, 25]. Even less data and evidence exist on the radiation response of cells resistant to hormonal therapy, which we believe is important considering the great incidence of resistance to systemic therapy in patients with breast cancer. In their study, Paulsen and colleagues investigated the influence of radiation on different breast cancer cell lines including cells resistant to tamoxifen (MCF-7/TAM^R^-1). The results of the study showed that the MCF-7/TAM^R^-1 cells were more resistant to ionizing radiation than the MCF-7 and MDA-MB-231 cell lines [22].

In this study, we analyzed gene expression changes during radiation responses in MCF-7 breast adenocarcinoma cells (MCF-7/S0.5) and in the tamoxifen resistant cell line MCF-7/TAM^R^-1 and the faslodex resistant cell line MCF-7/182^R^-6 derived from the MCF-7/S0.5 cell line. For the first time, we have shown that MCF-7/TAM^R^-1 cells have an elevated potential to withstand radiation-induced DNA damage and display a decreased sensitivity to ionizing radiation.

## RESULTS

### The effects of radiation on whole-genome gene expression in antiestrogen-sensitive and antiestrogen-resistant MCF-7 cells

**Figure 1:**
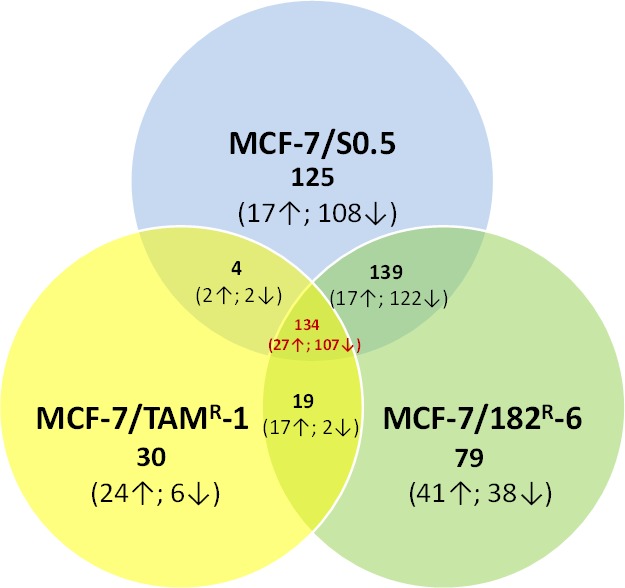
Gene expression profiling of MCF-7/S0.5, MCF-7/TAM^R^-1 and MCF-7/182^R^-6 The Venn diagram shows the number of significantly changed genes in the MCF-7/S0.5, MCF-7/TAM^R^-1 and MCF-7/182^R^-6 cell lines upon radiation in comparison to their corresponding un-irradiated controls, as identified by the gene expression profiling analysis. The arrows beside the numbers in brackets represent the direction of genes alteration (up- or down-regulation).

**Figure 2:**
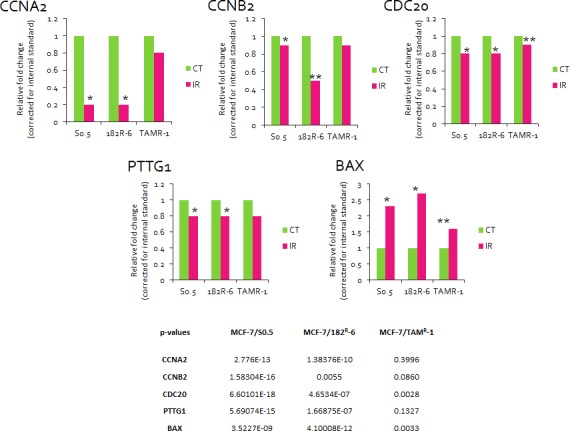
Fold change in the levels of *CCNA2, CCNB2, CDC20, PTTG1* and *BAX* transcripts detected by qRT-PCR Each treatment group was compared to its corresponding control. Actin was used as a reference gene (calculated by Pfaffl). * - significant, p<0.001; ** - significant, p<0.01. (Student's t-test).

The gene expression analysis was conducted for MCF-7/S0.5 and the antiestrogen-resistant derivatives, MCF-7/TAM^R^-1 and MCF-7/182^R^-6, with the purpose to evaluate and compare the radiation response between cell lines. Differential gene expression in the MCF-7 cell lines was found upon exposure to radiation. In fact, the expression level of 402, 371 and 187 genes was significantly altered due to X-ray exposure in MCF-7/S0.5, MCF-7/182^R^-6 and MCF-7/TAM^R^-1, respectively (Fig.1). Interestingly, most of the altered genes were down-regulated. Amongst 134 genes that were common for all three cell lines, 27 genes were up-regulated and 107 genes were down-regulated. The majority of gene expression changes observed in the antiestrogen resistant cell lines were also seen in the parental MCF-7/S0.5, (73.6 and 73.8% of the genes in MCF-7/182^R^-6 and in MCF-7/TAM^R^-1, respectively). The least gene expression changes were found in the MCF-7/TAM^R^-1 cell line which had only half as many gene changes as the parental and ICI 182,780 resistant cells, and only 30 unique genes changes in response to radiation treatment (Fig.1). Further, we uploaded the gene lists consisting of 402, 371 and 187 genes from the MCF-7/S0.5, MCF-7/182^R^-6 and MCF-7/TAM^R^-1 lines, respectively, through the DAVID pathway-specific enrichment analysis in order to identify casual relationships between the genes and organize them into specific pathways according to their functions. Subsequently, the genes with similar or identical functions were grouped together and organized by the KEGG database into pathways. The least number of genes that could constitute a pathway was three; therefore, only 83 genes and the 12 pathways they belong to were further studied (Suppl Table 1). Mainly, those were the genes that play a role in cell cycle, DNA replication, base excision repair (BER), nucleotide excision repair (NER), mismatch repair (MMR), homologous recombination (HR), p53 signaling, gap junction, drug metabolism, purine and pyrimidine metabolism and spliceosome. Based on each gene's function and its expression trend, the roles of the above-mentioned pathways were evaluated and compared between cell lines (Suppl Table 1 and Table 1). For this, the pathways were deemed significantly altered if at least 80% of the genes from the pathway were shifting the pathway in the same direction (Table 1) [26]. For instance, in the MCF-7/S0.5 line, eight out of ten genes from the p53 signaling pathway represented in Suppl Table 1 were changed in a way that functionally shifted the pathway to the overall up-regulation. These eight genes represented 80% of pathway significance in the MCF-7/S0.5 line, which allowed us to conclude that the p53 signaling pathway was significantly up-regulated in the MCF-7/S0.5 cells upon exposure to radiation (Table 1). An identical analysis approach was applied for the remaining 11 pathways in each cell line. Table 1 demonstrates the pathways' specific differences between MCF-7/S0.5, MCF-7/182^R^-6 and MCF-7/TAM^R^-1 in response to X-ray radiation (Table 1). As expected, 5 Gy of X-ray caused cell cycle deregulation in all three MCF-7 cell lines (Suppl. Fig. 1). The down-regulation in the expression level of 18 genes involved in cell cycle was common for MCF-7/S0.5, MCF-7/TAM^R^-1 and MCF-7/182^R^-6. These genes constituted the components of the mitotic checkpoint *CHEK, MAD2L1, BUB1* and *BUB1B, E2F* transcription factor 2, *CCNA2* and *CCNB2* encoding cyclins A2 and B2, cyclin-dependant kinase *CDC20*, the components of the minichromosome maintenance (MCM) complex, protein-kinase *TTK*, protease *ESPL11* and a regulator of chromosome stability *PTTG1*. In addition, MCF-7/S0.5 and MCF-7/182^R^-6 shared the down-regulation of *RAD2, CDC25C, CDC7, CDK2* and a negative regulator of entry into mitosis *PKMYT.* Both antiestrogen-resistant cell lines overexpressed growth arrest and *GADD45A,* a DNA-damage-inducible factor, upon radiation treatment (Suppl Table1). The second pathway that like the cell cycle was mostly affected by ionizing radiation in all cell lines was DNA replication. 20, 16 and 9 genes involved in the process of DNA replication were down-regulated in MCF-7/S0.5, MCF-7/182^R^-6 and MCF-7/TAM^R^-1, respectively (Table 1). Specifically, they were components of the minichromosome complex (*MCM 2-7*), DNA polymerases A, D and E, replication factors *RFC 2, 3, 4,* and *5*, the replication protein *RPA3* and others (Table 1). Moreover, the main DNA repair pathways were also downregulated in MCF-7/S0.5 and MCF-7/182^R^-6 in response to 5 Gy of X-rays. Base excision repair, mismatch repair, and homologous recombination were down-regulated in MCF-7/S0.5 and MCF-7/182^R^-6; and nucleotide excision repair (NER) was significantly down-regulated in MCF-7/S0.5 (Suppl Table 1 & Table 1). Moreover, the purine and pyrimidine metabolism pathways that could contribute to DNA replication and DNA repair by providing the necessary deoxyribonucleotides were also down-regulated in response to X-ray radiation. An inability of cells to ultimately replicate and repair their DNA leads to cell death. The P53 signaling pathway was functionally up-regulated in MCF-7 sensitive and antiestrogen-resistant cell lines in response to exposure to radiation (Table 1). The decreased expression of tubulins, the main components of microtubules, resulted in the overall down-regulation of the gap junction pathway in MCF-7/S0.5 and MCF-7/182^R^-6 cells which could contribute to the apoptotic response; the down-regulation of spliceosome in MCF-7/182^R^-6 is translated into the absence of RNA processing that is necessary for protein synthesis and cell proliferation. Interestingly, an increase in the expression state of genes that contribute to drug metabolism was observed in the MCF-7/TAM^R^-1 cell line after radiation treatment. These genes were: flavin- containing monooxygenase (*FMO5*), glutathione S-transferase kappa 1 (*GSTK1*) and monoamine oxidase A (*MAOA*) that could potentially increase drug-resistance of MCF-7/TAM^R^-1 cells. Overall, although the radiation response of the three MCF-7 cell lines was similar in the way that all cells showed down-regulation of cell cycle, DNA replication, DNA repair and activation of the apoptotic pathway, the most dramatic response was found in the antiestrogen sensitive MCF-7/S0.5 cell line. The cells resistant to ICI 182,780 were also very sensitive to radiation, while tamoxifen-resistant cells showed the least dramatic response. Moreover, the up-regulation of the drug metabolism pathway post-radiation exposure suggests a possible strengthening of drug resistance by ionizing radiation in MCF-7/TAM^R^-1 cells. The gene expression data have been confirmed by the qRT-PCR analysis on the five genes that play a role in the cell cycle and apoptosis: *CCNA2* and *CCNB2*, *CDC20*, *PTTG1* and *BAX*. Similarly to the gene expression data, qRT-PCR showed a significant down-regulation of *CCNA2, CCNB2, CDC20, PTTG1* and up-regulation of *BAX* in the three MCF/7 cell lines 24 hours after radiation exposure (Fig.2).

**Table:** 

Pathway	MCF-7/S0.5	MCF-7/182^R^-6	MCF-7/TAM^R^-1
BER	−100% (9)	−100% (7)	-
Cell cycle	−100% (25)	−100% (25)	−100% (19)
DNA replication	−100% (20)	−100% (16)	−100% (9)
Drug metabolism	-	-	+100% (3)
Gap junction	−100% (8)	−100% (7)	-
HR	−100% (6)	−100% (4)	-
MMR	−100% (7)	−100% (6)	-
NER	−81.8% (11)	N/S (9)	N/S (4)
P53 signaling	+80% (10)	+84.6% (13)	+88.95 (9)
Purine metabolism	−90.9% (11)	-	-
Pyrimidine metabolism	−92.3% (13)	−87.5% (8)	−100% (5)
Spliceosome	-	−100% (7)	-

### Radiation-induced DNA damage in MCF-7/S0.5, MCF-7/182^R^-6 and MCF-7/TAM^R^-1

**Figure 3:**
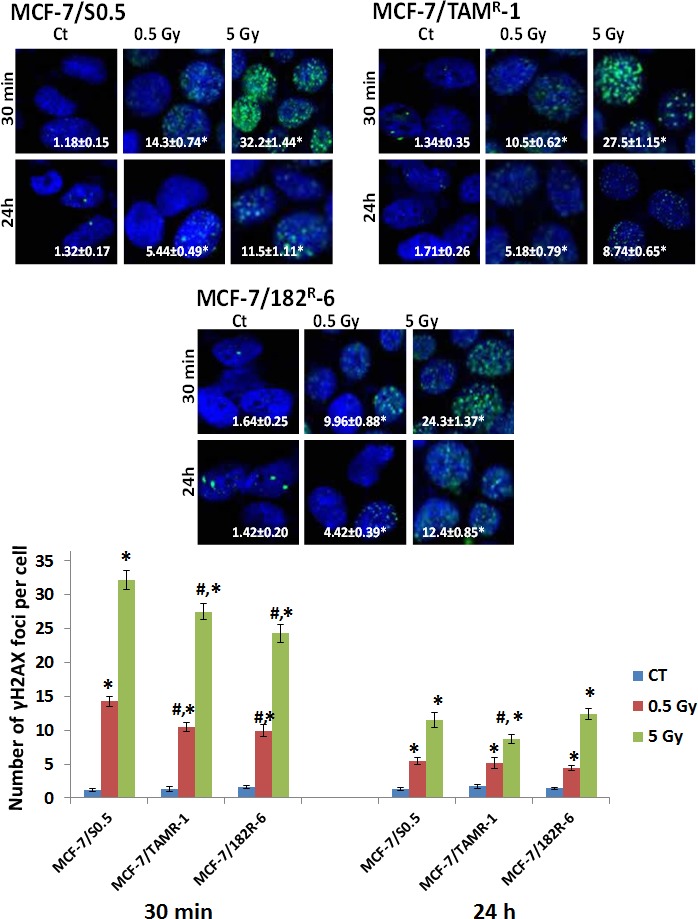
Radiation-induced H2AX phosphorylation in MCF-7/S0.5, MCF-7/TAM^R^-1 and MCF-7/182^R^-6 cells The results on the pictures and a figure below are presented as an average number of γH2AX foci per cell ± SE, n = 200. * - significantly different from the respective control; p < 0.05; # - significantly different from MCF-7/S0.5 cell line. Controls were not significantly different between three cell lines; p<0.05. Magnification, × 100. Blue – DAPI, green – γH2AX.

The gene expression changes found in the three MCF-7 lines, MCF-7/S0.5, MCF-7/182^R^-6 and MCF-7/TAM^R^-1, were accompanied with the extensive DNA damage caused by radiation. Ionizing radiation (IR) is a potent DNA-damaging agent capable of inducing cross-linking, nucleotide base damage, and most importantly, single- and double-strand breaks (DSBs) which are well-known inducers of apoptosis [27, 28]. Therefore, we analyzed and compared the levels of IR-induced DNA damage in MCF-7/S0.5, MCF-7/182^R^-6 and MCF-7/TAM^R^-1 cells by detecting γH2AX foci, a well accepted indicator of DNA double-strand breaks [29] and by the Comet assay. To better study the dynamics of the appearance of γH2AX foci in MCF-7 breast cancer cells, we added another time point (30 minutes) and a lower IR dose (0.5 Gy) to the already existing experimental conditions. As expected, the appearance of γH2AX foci in all three cell lines was dose-, and time-dependant. Both the intermediate (0.5 Gy) and high (5 Gy) doses of X-rays caused a significant elevation in the level of γH2AX foci in antiestrogen-sensitive and antiestrogen-resistant cells (Fig.3). The highest γH2AX level was observed at the 30-minute time point. Specifically, 12.1-, 7.84-, and 6.07-fold changes compared to controls were caused by 0.5 Gy; and 27.3-, 20.5-, and 14.8-fold changes were caused by 5 Gy of X-rays 30 minutes after exposure in MCF-7/S0.5, MCF-7/TAM^R^-1 and MCF-7/182^R^-6, respectively (Fig.3). Here, it is important to note that 30 minutes after exposure to 0.5 and 5 Gy of X-rays both antiestrogen-resistant cell lines accumulated significantly less DSBs than their antiestrogen-sensitive parental line MCF-7/S0.5 line. Approximately a halfway decrease in the level of γH2AX foci was achieved from the 30-min to 24-h time point in all three cell lines indicating DNA repair and/or damage-induced apoptosis during this period. Therefore, at the 24-hour time point, the level of foci was different from that in the control non-radiated cells by 4.12-, 3.03-, and 3.11-fold for the 0.5 Gy dose and by 8.71-, 5.11-, and 8.73-fold for the 5 Gy dose of X-rays in MCF-7/S0.5, MCF-7/TAM^R^-1 and MCF-7/182^R^-6, respectively (Fig.3). Interestingly, MCF-7/TAM^R^-1 cells displayed more complete repair of IR-induced DNA damage than the other two lines 24 hours after exposure to 5 Gy of X-rays. The number of γH2AX foci in tamoxifen-resistant MCF-7/TAM^R^-1 cells at this time point was significantly lower than in other cell lines. Overall, the immunofluorescent analysis showed that the background level of γH2AX foci was similar for the three cell lines, and the induction of foci by radiation had a similar trend between the MCF-7/S0.5 cell line and the two anti-estrogen-resistant cell lines, MCF-7/TAM^R^-1 and MCF-7/182^R^-6. Nevertheless, MCF-7/S0.5 cells displayed significantly higher level of DNA DSBs after each applied dose in comparison to the antiestrogen-resistant cells. Additionally, MCF-7/TAM^R^-1 cells were able to repair IR-induced damages 24 hours after irradiation more efficiently than the other two lines.

**Figure 4:**
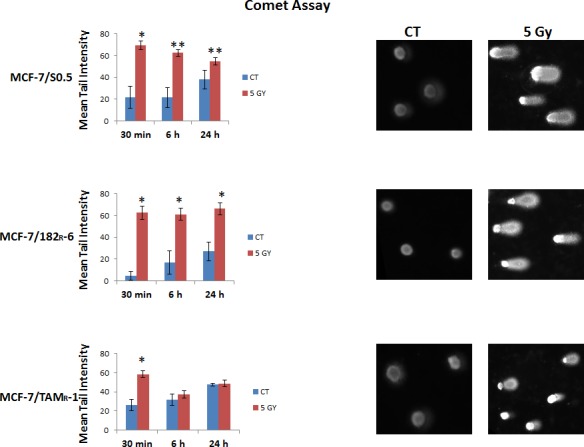
Radiation-induced DNA damage in MCF-7/S0.5, MCF-7/TAM^R^-1, and MCF-7/182^R^-6 cells as determined by the Alkaline Comet assay The graphs represent the percentage of DNA in the comet tails (tail intensity) obtained by the Alkaline Comet assay performed on MCF-7/S0.5, MCF-7/TAM^R^-1, and MCF-7/182^R^-6 cells 30 minutes, 6 and 24 hours after X-ray irradiation. Tail intensity levels are represented as mean ± SD; * - significantly different from the respective control, p < 0.01; ** - significantly different from the respective control, p<0.05. (Student's t-test). Comet representative pictures of tail intensity are located beside the charts.

In the comet assay, the super coiled duplex DNA underwent unwinding and denaturation under strong alkaline conditions [30]. This led to the reduction of DNA fragment size and the expression of alkali labile sites as single-strand breaks which are stretched out by electrophoresis. A comet tail consisting of the broken or damaged DNA fragments was analyzed through the intensity in MCF-7/S0.5, MCF-7/TAM^R^-1 and MCF-7/182^R^-6 cells after radiation treatment (Fig.4). A 5 Gy X-ray treatment led to significant damage in MCF-7 parental and both drug resistant cells immediately (30 min) after the application. These damages are believed to represent DSBs, SSBs, alkali labile sites, and breaks from replication events. But the persistence of damages was only observed in MCF-7/S0.5, and MCF-7/182^R^-6 cells at the 6- and 24-hour time points, and no significant damages were observed in the drug-resistant line MCF-7/TAM^R^-1 (Fig.4). Such difference could be associated with a higher potential for DNA repair in cells resistant to tamoxifen.

### Radiation-induced apoptosis in MCF-7/S0.5, MCF-7/TAM^R^-1 and MCF-7/182^R^-6 cells

**Figure 5:**
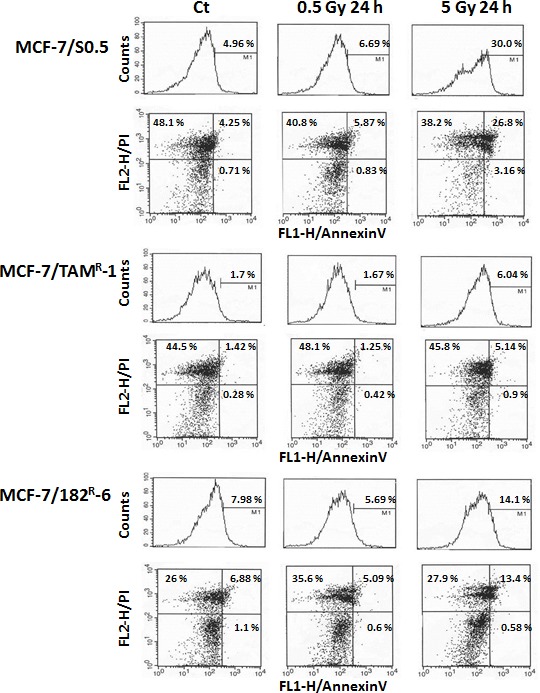
Radiation-induced apoptosis in MCF-7/S0.5, MCF-7/TAM^R^-1 and MCF-7/182^R^-6 cells The number of cells in early apoptosis was measured using the Annexin V-FITC assay for control cells (CT) and cells irradiated with 0.5 Gy and 5 Gy of X-rays. M1 – AnnexinV- positive cells; Viable cells - AnnexinV- and PI-negative (the lower left quadrants); Cells in the early apoptosis state - AnnexinV-positive and PI-negative (the lower right quadrants); Cells in the late apoptosis state or already dead cells - both Annexin V- and PI-positive (the upper right quadrants).

IR exposure is known to induce apoptotic cell death. Therefore, we analyzed the levels of IR-induced apoptosis in MCF-7/S0.5 and two antiestrogen-resistant lines, MCF-7/182^R^-6 and MCF-7/TAM^R^-1. Early apoptosis is characterized by various changes in the cellular plasma membrane; the primary change is the translocation of phosphatidylserine (PS) from the inner layer to the surface of the membrane. Annexin V possesses a high affinity to PS, and this allows for the early detection of apoptotic changes [31]. Here, we analyzed IR-induced apoptosis using an Annexin V assay for MCF-7 breast adenocarcinoma cells 24 h post radiation exposure. Interestingly, 0.5 Gy of X-rays did not cause any significant changes in the level of early apoptosis in either of cell lines. In contrast, 5 Gy X-rays led to a significant apoptosis in all three cell lines (Fig.5). The percentage of annexin V-positive cells increased from 4.96 % to 30.0 % in MCF-7/S0.5; from 7.98% to 14.1 % in MCF-7/182^R^-6; and from 1.7 % to 6.04 % in MCF-7/TAM^R^-1 at 5 Gy of irradiation at 24 hours post radiation (Fig.5). Overall, the annexin V assay showed that the antiestrogen-sensitive MCF-7/S0.5 line is more sensitive to radiation-induced apoptosis than the antiestrogen-resistant MCF-7/182^R^-6 and MCF-7/TAM^R^-1 lines.

## DISCUSSION

The effect of systemic therapy on patients with breast cancer has been widely debated. A variety of alternatives for breast cancer treatment are constantly expanding but the combination of chemotherapy, radiation therapy, surgery and hormone therapy for the appropriate treatment plan is still complex [32]. Although these therapies have proven to be beneficial, a large number of patients acquire resistance to treatments.

The purpose of this study was to investigate radiation-induced gene expression changes in three cell lines of breast adenocarcinoma: the parental MCF-7/S0.5 and the antiestrogen- resistant MCF-7/TAM^R^-1 and MCF-7/182^R^-6. Using microarray technology tools, we were able to screen differences in gene expression in response to radiation between MCF-7/S0.5, MCF-7/TAM^R^-1 and MCF-7/182^R^-6. Here, we show that these three cell lines respond differently to radiation at the gene expression level. Gene expression profiling showed that the expression level of at least 402 and 371 genes changed in the antiestrogen-sensitive MCF-7/S0.5 cell line and in cells resistant to ICI 182,780, respectively, due to 5 Gy X-rays. However, in MCF-7/TAM^R^-1 cells, only 187 genes changed (Fig.1). We believe that the ability of cells to retain their gene expression potential at a close to constant level regardless of DNA-damaging insults may be due to some features acquired by antiestrogen-resistant cells and shared in other forms of resistance, such as radiation resistance. Interestingly, most of the changed genes were down-regulated in all three cell lines. Using David software, we have revealed that these genes belong mainly to the cell cycle, DNA replication and DNA repair pathways (Suppl Table 1). The most profound down-regulation of gene expression was observed in genes involved in the cell cycle pathway (Table 1, Suppl. Fig.1). The reduced expression of the S and M cyclins, cyclin A2 and B2 (Fig.2), and their cyclin-dependant kinase CDK2 indicate cell cycle arrest in S or G2/M phases of the cell cycle (Suppl Table 1, Suppl. Fig.1). Moreover, the similar down-regulation of the E2F transcription factor may prevent cells from entering the S-phase. In addition, the lower expression of PTTG1, the TTK protein kinase that is usually present in rapidly proliferating cells, (Fig.2) that peaks in the M phase, ORC3L that binds to origins of replication, CDC7, one of the regulators of the G1/S transition, CDC25C, an inducer of mitotic control that is necessary for cell cycle progression, and CDC20 (Fig.2), an activator of APC and a major regulator of cell division, reflects cell cycle disturbance in all three cell lines. One would expect that the cells were arrested at the cell cycle checkpoints, but surprisingly, most of the mitotic checkpoint regulators were also down-regulated. Among them were: CHEK1 that phosphorylates the components of CDC25 for cell cycle arrest; MAD2 that interacts with CDC20 and is a component of the spindle-assembly checkpoint that prevents anaphase until chromosomes are correctly aligned, and BUB1 that is involved in cell cycle checkpoint enforcement (Suppl Table1). These gene expression data represent the total cell-cycle shutdown and checkpoint failure which are most probably due to extensive DNA damages caused by ionizing radiation. Cell cycle checkpoints usually contribute to cell survival allowing for DNA damage repair; and the lack of checkpoints makes cells more sensitive to killing by ionizing radiation [33].

Both the cell cycle and DNA replication pathways shared the common down-regulation of six components of the minichromosome maintenance complex (MCMs: 2, 3, 4, 5, 6, 7) in all three cell lines (Suppl Table 1, Suppl. Fig.1). The MCM 2-7 helicase complex is important for the replication fork formation and elongation during DNA replication [34]. In fact, it is required for the assembly of pre-replication complexes (pre-RCs) at replication origins at the end of mitosis and during late G1 [35, 36]. It is evident that mammalian cells decrease the rate of ongoing DNA synthesis in response to DNA damage at the level of origin initiation and fork progression [37]. Obviously, the inactivation of the MCM complex inhibits DNA replication and cell proliferation and can be the mechanism of cell cycle arrest. Indeed, the down-regulation of MCM2 and MCM6 was associated with Notch-dependant cell cycle arrest in endothelial cells and human fibroblasts [38]. In response to genotoxic stress such as ionizing radiation, the ATM/ATR checkpoint pathways are activated and target stalled replication forks. The MCM complex is also a target of checkpoint signaling [39]. Stalled replication forks must retain MCM proteins in order to resume replication. Otherwise, replication licensing cannot be reassembled as origins fire only once in each cell cycle [36]. The down-regulation of MCM 2-7 in MCF-7/S0.5, MCF-7/TAM^R^-1 and MCF-7/182^R^-6 (Suppl Table 1) in response to X-ray radiation indicates aberrant DNA replication or its absence and cell cycle arrest. In addition, reduced expression levels of DNA polymerases add up to the disruption of DNA replication and/or repair. Here, it is important to emphasize that mainly DNA polymerases from MCF-7/S0.5 and MCF-7/182^R^-6 are inhibited, and only polymerase PolE2 is also effected in MCF-7/TAM^R^-1 (Suppl Table 1). The other necessary components of the DNA replication/repair pathway which were down-regulated in response to ionizing radiation were: LIG1 (a ligase that seals nicks in double-stranded DNA during replication, recombination and repair), PRIM1 (a primase that synthesizes short RNA primers for Okazaki fragments during discontinuous replication), FEN1 (an endonuclease that cleaves the 5′-overhanging flap structure that is generated by displacement synthesis when DNA polymerase encounters the 5′-end of a downstream Okazaki fragment), RNASEH2A (a ribonuclease that removes RNA primers from lagging-strand Okazaki fragments), RFC 2-5 (replication factors that play a role of a clamp loader for loading PCNA on DNA during replication), and RPA3 (the replication protein that binds ssDNA and keeps it unwound for DNA replication or repair). Overall, 20, 16 and 9 DNA replication genes were down-regulated in MCF-7/S0.5, MCF-7/182^R^-6 and MCF-7/TAM^R^-1, respectively. Furthermore, the detected down-regulation of purine and pyrimidine metabolism mainly in MCF-7/S0.5 and MCF-7/182^R^-6 contributes to the decreased DNA replication/repair. The importance of sufficient nucleotide pools in the S phase is reflected by the G1 arrest when the pools are inadequate [40]. Further evaluation of genes by functional relationships with pathways showed the similarity in the radiation response between MCF-7/S0.5 and MCF-7/182^R^-6 (Table 1). Both cell lines exhibited a lower expression of DNA repair genes following radiation exposure. Specifically, the down-regulation of base excision repair, nucleotide excision repair, mismatch repair and homologous recombination was observed. In addition to the previously mentioned genes (DNA polymerases, RFCs, RPAs, FEN1 and LIG1) that clearly participate in DNA repair, some specific repair genes were also down-regulated (Suppl Table 1). These genes were the following: uracil-DNA glycosylase (UNG that excises uracil residues from DNA that can arise as a result of misincorporation of dUMP residues by DNA polymerase or due to the deamination of cytosine); poly(ADP-ribose) polymerase 2 (PARP2 that catalyzes the poly ADP-ribosylation of a limited number of acceptor proteins involved in chromatin architecture and DNA metabolism) and high-mobility group box1-like1 (HMGB1L1 that binds preferentially single-stranded DNA and unwinds double-stranded DNA) in BER; mutS homolog 6 (MSH6 that heterodimerizes with MSH2 to form MutS alpha that binds to DNA mismatches, thereby initiating DNA repair) in MMR; Bloom syndrome, REcQ helicase-like (BLM that unwinds single- and double-stranded DNA in a 3′-5′ direction); RAD51 homolog C (RAD51C that is involved in the homologous recombination repair pathway of double-stranded DNA breaks arising during DNA replication or induced by DNA-damaging agents); RAD54-like (RAD54L that is involved in DNA repair and mitotic recombination) and X-ray repair complementing defective repair in Chinese hamster cells 3 (XRCC3 that is thought to repair chromosomal fragmentation, translocations and deletions) in HR. Interestingly, two genes involved in NER, damage-specific DNA binding protein (DDB2) and xeroderma pigmentosum complementation group C (XPC) involved in DNA damage recognition and initiation of DNA repair were up-regulated in MCF-7/S0.5 and MCF-7/182^R^-6. This might mean that DNA damages are initially recognized, but the actual repair failed due to the lack of downstream components of the pathway. Such results demonstrate that radiation-induced DNA damages (especially in MCF-7/S0.5 and MCF-7/182^R^-6) are too great for cell survival and lead to DNA repair failure and possibly to cell death. In contrast, there were no significant changes in the expression level of DNA repair genes in MCF-7/TAM^R^-1 cells. The immunocytochemical staining of cells for γH2AX proved the radiation-induced formation of DNA damages, specifically DSBs, and the initiation of DNA repair in all three cell lines. The induction of the DSBs was dose- and time-dependant (Fig.3). Although many DSBs were repaired in 24 hours, the level of γH2AX never returned to the initial one. At the 24-hour time point, a lot of DSBs caused by both low and high doses remained unrepaired in all three cell lines. Interestingly, MCF-7/TAM^R^-1 cells displayed significantly lower levels of γH2AX foci at 24 hours upon exposure to 5 Gy of X-rays in comparison to the other two cell lines that were shown to be DNA repair defective in gene expression analysis. Considering, that γH2AX staining only detects DSB damages in DNA, we performed the Comet assay to evaluate the broader types of damages. These damages are believed to represent DSBs, SSBs, alkali labile sites, and breaks from replication events. Although, all three cell lines displayed a rapid increase (30 minutes) in the levels of radiation-induced DNA damage, MCF-7/TAM^R^-1 cells showed no significant persistence of DNA damages (Fig.4). 6 and 24 hours after radiation exposure, the level of DNA damages represented by the comet tail intensity was similar to the control level in MCF-7/TAM^R^-1 cells. In contrast, the level of DNA damages in MCF-7/S0.5 and MCF-7/182^R^-6 cells remained high even at 24 hours post radiation. These data suggest that MCF-7/TAM^R^-1 cells have a higher DNA repair activity after radiation in comparison to MCF-7/S0.5 and MCF-7/182^R^-6 cells. The ability to withstand and repair DNA damage may result in reduced sensitivity to radiation and possibly demands other types of cancer treatment.

The majority of DNA damage signaling proteins may be inactivated by caspases during the execution phase of apoptosis [41]. P53 is one of the main executioners of cellular response to ionizing radiation and apoptosis. Its levels are elevated in response to ionizing radiation affecting a number of downstream effector genes, such as Bax, p21, GADD45G and Mdm2 [41]. Radiation-induced p53 activation causes the cell cycle arrest allowing for DNA repair and in the case of repair failure, p53 triggers apoptosis [42]. In agreement with the above, p53 signaling was activated in all three cell lines in response to radiation. Up-regulated BAX (Suppl Table 1, Fig.2) is known to accelerate programmed cell death by binding and inhibiting an apoptosis repressor Bcl-2. The activation of sestrin 1 (Suppl Table 1) was previously shown upon genotoxic exposure, and its cytoprotective function based on regeneration of overoxidized peroxiredoxins was described [43]. A few years ago, Budanov and Karin showed that sestrin is a target of p53 and an inhibitor of TOR (target of rapamycin). mTOR is a phosphatidylinositol kinase-related kinase that positively regulates cell growth. P53-mediated activation of sestrin upon genotoxic stress inhibits mTOR through the AMP-responsive protein kinase (AMPK) [44]. Gene activated by p53, the ribonucleotide reductase (RRM2B), was up-regulated in MCF-7/S0.5 and MCF-7/182^R^-6. RRM2B plays a role in DNA repair of arrested cells by supplying deoxyribonucleotides during cell cycle arrest in a p53-dependent manner. Although it is not clear whether this gene actually affected DNA repair, considering the fact that its homolog RRM2 that also provided precursors for DNA synthesis was down-regulated in all three cell lines). Finally, the increased expression of Gadd45A and TP53I3 in antiestrogen-resistant cells also indicate the cell cycle arrest after X-ray treatment (Suppl Table 1). The gene expression data correlate with the results of the annexin V assay on early apoptosis. Exposure to 5 Gy of X-rays initiated apoptotic cell death in all three cell lines. However, the degree of apoptosis was different in between the cell lines. The highest apoptosis level was detected in MCF-7/S0.5 cells (30%), while in cells resistant to tamoxifen, only 6% of the cells were undergoing apoptosis (Fig.5). Such differences can be attributed to the radio-resistance of MCF-7/TAM^R^-1 cells. In fact, although the response to X-rays (such as an increase in DNA damages and cell cycle arrest) in MCF-7/TAM^R^-1 cells and the other two cell lines was similar, MCF-7/TAM^R^-1 cells did not lose their DNA repair capacity and exhibited lower fraction of apoptotic MCF-7/TAM^R^-1 cells compared to parental and ICI 182,780 resistant cells.

According to the gene expression profiling and the data of pathway enrichment analysis, a strong down-regulation of the gap junction pathway was caused by the ionizing radiation in MCF-7/S0.5 and MCF-7/182^R^-6 but not in MCF-7/TAM^R^-1 (Table 1). All down-regulated genes that constituted the pathway were members of cytoskeletal elements, tubulins alpha and beta. An altered level of expression of cytoskeletal elements plays a considerable role in radiation-mediated transformation. The differential modulation of genes encoding cytoskeletal elements upon radiation exposure was previously documented, where actin and tubulin mRNA accumulation was reported to be similar to that in transformed cells [45]. Cancer cells are characterized by a complicated ultrastructural organization. Breast cancer cells resistant to doxorubicin and cisplatin display an increase in the number of microtubules and varying widths of microfilaments [46]. Tubulins are critical for cell division, which made them a target for several anti-cancer drugs. An elevated expression of tubulin correlates with a lack of response to chemotherapy. In fact, βIII-tubulin expression has been acknowledged as a predictor of the docetaxel resistance in metastatic prostate cancer [47]. Another study claimed that βII-tubulin is a strong predictor of outcome in patients treated with the platinum-based induction chemotherapy for locally advanced squamous carcinoma of the head and neck [48]. A possible explanation for such observation was based on the fact that tubulin binds to the voltage-dependent anion channel, VDAC, and regulates the permeability of the mitochondrial outer membrane. Binding of tubulin to VDAC inhibits the binding of proapoptotic drugs which induce a rapid cytochrome c release [48]. In the present study, a decrease in tubulin expression in the MCF-7/S0.5 and MCF-7/182R-6 cell lines indicates the inability of the formation of a tubular apparatus necessary for cell division, and it also supports the data on early apoptosis. In contrast, MCF-7/TAM^R^-1 cells did not show any expression changes in a single tubulin gene, which at least partly may contribute to the reduced sensitivity to radiation.

In addition, three genes involved in drug metabolism were up-regulated in MCF-7/TAM^R^-1 cells. One of these genes was glutathione S-transferase kappa 1 (GSTK), a radical scavenger that is involved in the metabolism of xenobiotics. It was previously found that GST plays an important role in the acquisition of drug resistance through the decreased intracellular drug accumulation and the stimulation of drug-induced DNA damage repair [49, 50]. Using an *in vivo* mouse model, it has been shown that tamoxifen-resistant tumors had a statistically significant increase in GST activity, the increased levels of other antioxidant enzymes such as SOD, and the reduced glutathione levels [51]. The authors discussed the effects of tamoxifen on the intracellular redox status of breast cancers, the induction of lipid peroxidation and the activation of antioxidant enzymes. Such oxidative changes appeared to be tamoxifen-specific as they were not found in ICI-resistant tumors [51]. In a recent study, a quantitive proteomic analysis revealed up-regulation of GST in breast cancer cells during the transition to acquired tamoxifen resistance [52]. Taking into consideration that ionizing radiation may also influence the redox status of cells, we believe that GST may be involved in the resistance of cancer cells to radiation, and therefore, may be considered one of the common molecular indicators for chemo- and radio-resistance. The second gene belonging to the drug metabolism pathway was flavin containing monooxygenase 5 (FMO). The protein product of this gene is an enzyme that belongs to the family of the enzymes involved in oxidation and metabolism of xenobiotics. This enzyme uses a flavin cofactor for its chemical reactions [53]. FMO enzyme system contributes to resistance to triclabendazole in liver fluke by metabolizing it to triclabendazole sulphooxide [54]. While flavin-containing monooxygenases were shown to convert tamoxifen to tamoxifen-N-oxide (TNO), TNO may be reduced back to tamoxifen by hemoglobin and cytochromes P450 [55]. The third gene in the up-regulated drug metabolism pathway was monoamine oxidase A (MAOA). MAOA product is an enzyme known to degrade amine neurotransmitters, such as dopamine, serotonine, epinephrine, and to cause severe depression, but was also shown to be involved in the metabolism of xenobiotics [56]. The up-regulation of the drug metabolism pathway in MCF-7/TAM^R^-1 cells after radiation treatment indicates that ionizing radiation may potentially decrease the sensitivity of tamoxifen resistant cells to xenobiotics and other treatment modalities (but not necessarily only cancer treatments).

Most recent studies have led to development of novel robust algorithms for transcriptome and pathway activation analysis. These may in turn be related to the potential responsiveness to chemotherapy agents. In the future it would be prudent to conduct transcriptome pathways profiling using these novels tools [57-59].

This study provides the analysis of the roles of DNA repair, and apoptosis in response to radiation in antiestrogen-sensitive and antiestrogen-resistant cell lines. The ability of tamoxifen-resistant cells to retain their DNA repair capacity upon radiation treatment allows us to suggest that DNA repair genes could possibly be considered as putative targets of the future development of novel anticancer regimens. Further detailed studies are needed to determine the cellular and molecular processes that are altered in resistant cells that allow them to survive genotoxic treatments such as irradiation.

## MATERIALS AND METHODS

### Cell lines and cell culture conditions

The MCF-7/S0.5 (MCF-7), MCF-7/TAM^R^-1 (TAM^R^-1) and MCF-7/182R-6 (182^R^-6) cell sublines were a kind gift from Anne Lykkesfeldt (Breast Cancer Group, Cell Death and Metabolism, Danish Cancer Society Research Center, DK-2100 Copenhagen, Denmark). Subline 0.5 derived from MCF-7 cells was originally adapted to grow on 0.5% fetal calf serum [60]. Tamoxifen and ICI 182,780 (fulvestrant, Faslodex) resistant sublines were derived from MCF-7/S0.5 as described previously [61, 62]. MCF-7/S0.5 cells were grown and maintained in Dulbecco's Modified Eagle's Medium (DMEM /F-12) with 2.5 mM L-Glutamine, without HEPES and Phenol Red (HyClone, Logan, UT), supplemented with 2% heat-inactivated fetal bovine serum (HyClone, Logan, UT) and 6 ng/ml of insulin (Sigma-Aldrich Chemical Co., St. Louis, MO) at 37 ºC in a 5% CO2 atmosphere. The MCF-7/TAM^R^-1 and MCF-7/182^R^-6 cell lines were grown in the identical medium as described above for the MCF-7/S0.5 line and were additionally supplemented with either 1 μM tamoxifen (Sigma-Aldrich) or 0.1 μM ICI 182,70 (Tocris Bioscience), respectively. Cells were harvested for analyses by trypsinization.

### Irradiation conditions

Cells were irradiated at 80% confluence in Dulbecco's Modified Eagle Medium (DMEM). Two radiation doses (0.5 Gy and 5 Gy, 90 kVp, 5 mA) were applied to check cellular radiation responses. Unirradiated cells served as controls. Cells were harvested 30 minutes and 24 hours after irradiation. All treatments were tested in triplicate. The experiments were independently reproduced twice.

### Whole-genome gene expression profiling

### RNA isolation

Total RNA was isolated using the Illustra RNAspin mini kit (GE Healthcare Life Sciences, Buckinghamshire, UK). Approximately 5 × 106 cultured cells were processed following the manufacturer's instructions. Samples were eluted in Ultrapure DNase/RNase-free distilled water provided in the kit. RNA samples were quantified using ultraviolet spectroscopy (NanoDrop, Wilmington, DE) and were further assessed for RNA integrity (RIN) on the Aglient 2100 Bioanalyzer (Santa Clara, CA) using the RNA Nano-chip Kit. RNA samples with RIN values of seven or higher were used for further analysis.

### Library preparation

cDNA was created using the Ambion's Illumina TotalPrep RNA Amplification Kit (Applied Biosystems, Carlsbad, CA) with an input of 500 ng of total RNA per sample. Briefly, oligo-dT primers were used to synthesize first-strand cDNA containing the phage T7 promoter sequence. Single-stranded cDNA was converted into a double-stranded DNA template via DNA polymerase. Simultaneously, RNase H degraded the RNA. Samples of cDNA were purified in the Filter Cartridge to remove excess RNA, primers, enzymes, and salts. The recovered cDNA was subjected to *in vitro* transcription using biotinylated UTPs. In this step, cRNA was created, labeled, and amplified. A final purification step removed unincorporated NTPs, salts, inorganic phosphates and enzymes, thus preparing samples for hybridization.

### Hybridization and detection

The Illumina's direct hybridization assay kit was used to process samples according to the manufacturer's protocol (Illumina, San Diego, CA). Overnight 750 ng of each cRNA sample were hybridized into the Illumina HumanHT-12_v4 Whole Genome Expression BeadChip arrays. A10-minute incubation in the supplied wash buffer at 55ºC preceded a 5-minute room temperature wash. The arrays were incubated in 100% ethanol for 10 minutes. A second room temperature wash lasted two minutes with gentle shaking, thus completing this high-stringency wash. The arrays were blocked with a buffer for 10 minutes and washed before a streptavidin-Cy3 (1:1000) probe for 10 minutes. After a five-minute wash at room temperature, the BeadChips were dried and imaged. Six controls were also built into the Whole-Genome Gene Expression Direct Hybridization Assay system to cover the aspects of array experiments, including controls for: the biological specimen (14 probes for housekeeping controls), three controls for hybridization (six probes for Cy3-labeled hybridization, four probes for low-stringency hybridization, and one probe for high-stringency hybridization), signal generation (two probes for biotin control), and approximately 800 probes for negative controls on an eight-sample BeadChip. The arrays were scanned on the iScan platform (Illumina), and the data were normalized and scrutinized using Illumina BeadStudio Software.

### BeadChip statistical analysis and data processing

The false discovery rate (FDR) was controlled using the Benjamini-Hochberg method. The Illumina Custom Model took FDR into account and was used to analyze the data. Differential gene expression (at least a 0.5-fold change) from non-radiated cells was determined to be statistically significant if the p value after the adjustment using the Benjamini-Hochberg method was lower than 0.05. The values were transformed to show a log2 scale.

Lists of regulated transcripts were inserted into the web-based DAVID Bioinformatics Resources 6.7 (NIAID/NIH) Functional Annotation Tool [46, 63]. This program was used to group genes into functionally relevant categories: metabolic processes, responses to stimulus/stress, DNA repair processes, apoptosis, and cell cycle processes. The minimum number of genes in each altered pathway has been set to three in order for a pathway to be considered for further evaluation. The pathways deemed significantly altered if at least 80% of genes were shifting the pathway in the same direction [26].

### Quantitative real-time PCR

Quantitative real-time PCR was performed to confirm the results of the Whole-Genome Gene Expression analysis for the regulation of the direction (either up or down) of selected genes. Five genes (CCNA2, CCNB2 CDC20, PTTG1 and BAX) were selected from the gene list of significantly differentially expressed transcripts representing a preliminary review of the acquired gene expression data. Actin was used as a reference gene. All reactions were performed using cDNA synthesized from the same RNA extraction as the BeadChip experiments, and 500 ng of the sample was used for the Bio-Rad iScript Select cDNA Synthesis Kit (Bio-Rad Laboratories, Hercules, CA). The samples were stored at −20ºC for long-term storage and at 4ºC until they were used for the subsequent qRT-PCR reactions.

Primers were designed using the NCBI database and PrimerQuest (Integrated DNA Technologies, Inc, Coralville, IA). The following primers were designed: the forward primer for the ACTA2 reference gene (5′-TAG CAC CCA GCA CCA TGA AGA TCA-3′) and the reverse primer (5′-GAA GCA TTT GCG GTG GAC AAT GGA-3′); CCNA2 forward primer (5′-ATG AGC ATG TCA CCG TTC CTC CTT-3′) and the reverse primer (5′-TCA GCT GGC TTC TTC TGA GCT TCT-3′); CCNB forward primer (5′-TGC TTC CTG CTT GTC TCA GAA GGT-3′) and the reverse primer (5′-CAT TCT TGG CCA TGT GCT GCA TGA-3′); CDC20 forward primer (5′-ATG CGC CAG AGG GTT ATC AGA ACA-3′) and the reverse primer (5′-CAT TTC GGA TTT CAG GCG CAT CCA-3′); PTTG1 forward primer (5′-AGT GGA GTG CCT CTC ATG ATC CTT-3′) and the reverse primer (5′-TCC AGG GTC GAC AGA ATG CTT GAA-3′); BAX forward primer (5′-TTT CTG ACG GCA ACT TCA ACT GGG-3′) and the reverse primer (5′- TGT CCA GCC CAT GAT GGT TCT GAT-3′). The reactions were prepared using 1 μL of diluted cDNA, 10 pmol/μL of each forward and reverse primer, and SsoFast EvaGreen Supermix (Bio-Rad Laboratories, Hercules, CA) prepared according to the manufacturer's instructions. The samples were prepared in triplicate and were run on the Bio-Rad C1000 Thermal Cycler equipped with the CFX96 Real-Time System. The qRT-PCR protocol consisted of denaturation at 95ºC for 2 minutes; 43 cycles of denaturation (95ºC, 5 seconds) and annealing/extension (55ºC, 5 seconds); and a final extension at 65ºC for 5 seconds. Annealing temperature optimization, melting curve analysis, and gel analysis of the amplicon were performed for every set of primers. To evaluate PCR efficiency, a standard curve was established using a series of cDNA dilutions. The data was captured and organized using Bio-Rad CFX Manager 2.1 software (Bio-Rad Laboratories, Hercules, CA).

### QRT-PCR statistical analysis

The quantification data from the Bio-Rad CFX Manager software were analyzed using the Pfaffl method in Microsoft Excel [49]. Graphs showing a fold change from the untreated cells were created, and transcript regulation directions (up- or down-regulation) were matched to the results of the Whole-Genome Gene Expression analysis.

### Immunofluorescence

For immunocytochemical analysis, cells were grown on two-well Lab-Tek chamber slides (Nulge Nunc International Corp., Naperville, IL) and irradiated. After irradiation, the cells were fixed in 4% paraformaldehyde in PBS, permeabilized with 70% ethanol, and washed in PBS containing 0.1% TRITON-X100. Blocking was done in 8% BSA in PBS. For immunocytochemical detection, the cells were incubated for two hours at room temperature using an anti-γH2AX (Ser 139) rabbit antibody (1:100, Cell Signaling Technology Inc., Danvers, MA). Afterwards, the cells were rinsed and incubated with a 1:500 diluted secondary antibody - goat anti-rabbit IgG Alexa Fluor 488 (Invitrogen Molecular Probes, Eugene, OR). Cell nuclei were counterstained with 0.1 mg/mL 4′,6-diamidino-2-phenylindole dihydrochloride (DAPI) (Sigma-Aldrich Chemical Co., St. Louis, MO). The slides were mounted with an anti-fade fluorescence medium prepared from 1,4-diazabicyclo[2.2.2]octane (DABCO), polyvinyl alcohol and glycerol and analyzed using a Zeiss epifluorescent microscope.

The number of γH2AX foci per cell was counted in at least 200 cells from each cell group, as previously described [50]. The levels of γH2AX were represented as the mean ± SE; P ≤ 0.05.

### Alkaline Comet Assay

The alkaline comet assay protocol was based on Olive and Bannath (2006) and Tice and Vasques (1995) at cometassay.com [64, 65]. The cells that were grown in cultures were trypsinised, collected in 15-ml tubes, and centrifuged for three min at 1000 g to form a pellet. Next, the pellet was washed three times with ice-cold phosphate-buffered saline (PBS) without −Ca2+ and −Mg2+. Finally, the cells were resuspended in their final concentration of 1000 cells per 1 μL of cell suspension in ice-cold PBS. The cell suspension was stored on ice during the course of subsequent procedures.

Ten microliters of cell suspension were mixed with 75 ul of 1% low melting point (LMP) agarose pre-heated to 40 ºC, mixed gently through pipetting up and down, and applied to a fully frosted microscope slide (VWR) that was pre-coated with normal melting point agarose. The agarose was overlaid with a cover slip and allowed to solidify for two to three minutes on ice. The removal of the cover slip was followed by an application of 85 ul of 1% LMP agarose pre-heated to 40 ºC in order to form a protective layer on the top of the layer containing the cell suspension. The cover slip was repositioned, and the slides were placed on ice to allow the agarose to solidify.

The cover slips were removed, and the slides were placed in a freshly prepared alkaline lysis solution (2.5 M NaCl, 100 mM Na2EDTA, 10 mM Tris base, 1% Triton, and 0.1% Sodium Lauroyl Sarcosine (pH 10.0) adjusted to 4 ºC), left overnight at 4 ºC, and protected from light. Following the lysis step, the slides were rinsed with a freshly prepared electrophoresis solution (300 mM, 2mM EDTA (pH>14)). Next, the slides were placed in an electrophoresis tank, covered with a thin layer (1-2 mm) of electrophoresis buffer, and left for 30 min to permit alkaline DNA unwinding. Electrophoresis was performed for 25 minutes at 0.7 V/cm. Each electrophoresis included slides that belonged to the same experimental time-point.

After the completion of electrophoresis, the slides were washed three times for five minutes in a neutralization buffer (0.4 M Tris (pH=7.5)). The slides were stained with SYBR gold dye (Invitrogen), the comets were viewed using a epifluorescence microscope (Zeiss), and the image information was collected using a Comet Assay IV system (Perceptive Instruments).

The statistical analysis was performed to obtain the tail intensity data using SPSS software (IBM) and according to recommendations on the statistical analysis of the Comet assay [37]. The data was collected from three replicate cell culture flasks, at two slides per flask, and 50 cells were examined on each slide. The median of the log tail intensity from 50 cells was evaluated per each slide followed by the calculation of the mean of two medians from two slides derived from one cell culture flask. Finally, the mean values were compared between three flasks representing each treatment point using a one-way ANOVA. The levels of tail intensity were represented as mean ± SD; P ≤ 0.05.

### The Annexin V assay

For the early detection of apoptosis, an Annexin V-FITC Apoptosis Detection Kit I (BD Biosciences, San Jose, CA) was used according to the manufacturer's protocol. Cells were grown and irradiated as previously described (Section 2.2). The analysis was performed 24 hours after exposure to radiation. Cells were harvested, washed with PBS, resuspended in a 1X binding buffer, stained with Annexin V and propidium iodide for 15 min at 25 ºC in the dark, and analyzed using flow cytometry within one hour at the Flow Cytometry Core Facility (University of Calgary, Calgary, AB). The results were represented as a percentage of gated Annexin V positive cells.

## SUPPLEMENTARY MATERIAL FIGURE AND TABLE




