# Supplementary material for: Altered radiation responses of breast cancer cells resistant to hormonal therapy
Source: Oncotarget. 2014 Dec 2;6(3):1678–94. doi: 10.18632/oncotarget.3188 (PMC4359324; doi:10.18632/oncotarget.3188)
Supplement: Supplementary file 1 [file oncotarget-06-1678-s001.pdf]

Altered radiation responses of breast cancer cells resistant to hormonal therapy

Supplementary Material

CHEK1	CHK1 checkpoint homolog (S. pombe)
E2F2	E2F transcription factor 2
MAD2L1	MAD2 mitotic arrest deficient-like 1 (yeast)
TTK	TTK protein kinase
BUB1	budding uninhibited by benzimidazoles 1 homolog (yeast)
BUB1B	budding uninhibited by benzimidazoles 1 homolog beta (yeast)
CDC20	cell division cycle 20 homolog (S. cerevisiae)
CCNA2	cyclin A2
CCNB1	cyclin B1
CCNB2	cyclin B2
ESPL1	extra spindle pole bodies homolog 1 (S. cerevisiae)
MCM2	minichromosome maintenance complex component 2
MCM3	minichromosome maintenance complex component 3
MCM4	minichromosome maintenance complex component 4
MCM5	minichromosome maintenance complex component 5
MCM6	minichromosome maintenance complex component 6
MCM7	minichromosome maintenance complex component 7
PTTG1	pituitary tumor-transforming 1; pituitary tumor-transforming 2

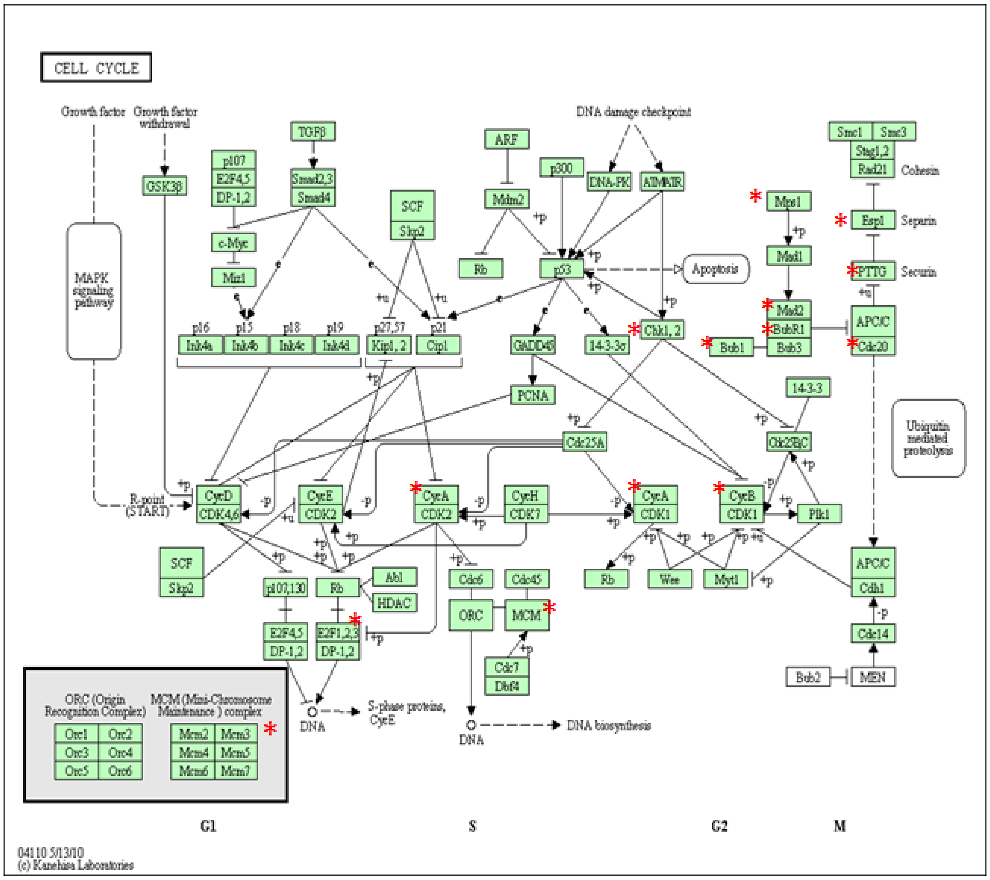

Supplementary Figure 1: The KEGG cell cycle pathway. The red stars indicate genes that were down-regulated in all three cell lines.
