# Supplementary material for: Altered radiation responses of breast cancer cells resistant to hormonal therapy
Source: Oncotarget. 2014 Dec 2;6(3):1678–94. doi: 10.18632/oncotarget.3188 (PMC4359324; doi:10.18632/oncotarget.3188)
Supplement: Supplementary file 2 [file oncotarget-06-1678-s002.pdf]

Supplementary Table-1

Gene symbol	*Gene name*	Cell line(s) where the gene expression is changed	KEGG Pathway(s)
FEN1-	*flap structure-specific endonuclease 1*	MCF-7/S0.5, MCF-7/182^{R}-6	DNA replication, BER
LIG1-	*ligase I, DNA, ATP-dependent*	MCF-7/S0.5	DNA replication, NER, BER, MMR
MCM2-	*minichromosome maintenance complex component 2*	MCF-7/S0.5, MCF-7/182^{R}-6, MCF-7/TAM^{R}-1	DNA replication, Cell cycle
MCM3-	*minichromosome maintenance complex component 3*	MCF-7/S0.5, MCF-7/182^{R}-6, MCF-7/TAM^{R}-1	DNA replication, Cell cycle
MCM4-	*minichromosome maintenance complex component 4*	MCF-7/S0.5, MCF-7/182^{R}-6, MCF-7/TAM^{R}-1	DNA replication, Cell cycle
MCM5-	*minichromosome maintenance complex component 5*	MCF-7/S0.5, MCF-7/182^{R}-6, MCF-7/TAM^{R}-1	DNA replication, Cell cycle
MCM6-	*minichromosome maintenance complex component 6*	MCF-7/S0.5, MCF-7/182^{R}-6, MCF-7/TAM^{R}-1	DNA replication, Cell cycle
MCM7-	*minichromosome maintenance complex component 7*	MCF-7/S0.5, MCF-7/182^{R}-6, MCF-7/TAM^{R}-1	DNA replication, Cell cycle
POLA1-	*polymerase (DNA directed), alpha 1, catalytic subunit*	MCF-7/S0.5	DNA replication, Pyrimidine metabolism, Purine metabolism

Supplementary Table-1

POLA2-	*polymerase (DNA directed), alpha 2 (70kD subunit)*	MCF-7/S0.5, MCF-7/182^{R}-6	DNA replication, Pyrimidine metabolism, Purine metabolism
POLD1-	*polymerase (DNA directed), delta 1, catalytic subunit 125kDa*	MCF-7/S0.5, MCF-7/182^{R}-6	DNA replication, NER, BER, Pyrimidine metabolism, Purine metabolism, MMR, HR
POLE-	*polymerase (DNA directed), epsilon*	MCF-7/S0.5	DNA replication, NER, BER, Pyrimidine metabolism, Purine metabolism
POLE2-	*polymerase (DNA directed), epsilon 2 (p59 subunit)*	MCF-7/S0.5, MCF-7/182^{R}-6, MCF-7/TAM^{R}-1	DNA replication, NER, BER, Pyrimidine metabolism, Purine metabolism
POLE4-	*polymerase (DNA-directed), epsilon 4 (p12 subunit)*	MCF-7/S0.5, MCF-7/182^{R}-6	DNA replication, NER, BER, Pyrimidine metabolism, Purine metabolism
PRIM1-	*primase, DNA, polypeptide 1 (49kDa)*	MCF-7/S0.5, MCF-7/TAM^{R}-1	DNA replication, Pyrimidine metabolism, Purine metabolism
RFC3-	*replication factor C (activator 1) 3, 38kDa*	MCF-7/S0.5, MCF-7/182^{R}-6	DNA replication, NER, MMR
RFC4-	*replication factor C (activator 1) 4, 37kDa*	MCF-7/S0.5, MCF-7/182^{R}-6, MCF-7/TAM^{R}-1	DNA replication, NER, MMR
RFC5-	*replication factor C (activator 1) 5, 36.5kDa*	MCF-7/S0.5, MCF-7/182^{R}-6	DNA replication, NER, MMR
RPA3-	*replication protein A3, 14kDa*	MCF-7/S0.5	DNA replication, NER, MMR, HR

Supplementary Table-1

RNASEH2A-	*ribonuclease H2, subunit A*	MCF-7/S0.5, MCF-7/182^R-6	DNA replication
CHEK1-	*CHK1 checkpoint homolog (S. pombe)*	MCF-7/S0.5, MCF-7/182^R-6, MCF-7/TAM^R-1	Cell cycle, p53
E2F2-	*E2F transcription factor 2*	MCF-7/S0.5, MCF-7/182^R-6, MCF-7/TAM^R-1	Cell cycle
MAD2L1-	*MAD2 mitotic arrest deficient-like 1 (yeast)*	MCF-7/S0.5, MCF-7/182^R-6, MCF-7/TAM^R-1	Cell cycle
RAD21-	*RAD21 homolog (S. pombe)*	MCF-7/S0.5, MCF-7/182^R-6	Cell cycle
SKP2-	*S-phase kinase-associated protein 2 (p45)*	MCF-7/S0.5	Cell cycle
TTK-	*TTK protein kinase*	MCF-7/S0.5, MCF-7/182^R-6, MCF-7/TAM^R-1	Cell cycle
BUB1-	*budding uninhibited by benzimidazoles 1 homolog (yeast)*	MCF-7/S0.5, MCF-7/182^R-6, MCF-7/TAM^R-1	Cell cycle
BUB1B-	*budding uninhibited by benzimidazoles 1 homolog beta (yeast)*	MCF-7/S0.5, MCF-7/182^R-6, MCF-7/TAM^R-1	Cell cycle
CDC20-	*cell division cycle 20 homolog (S. cerevisiae)*	MCF-7/S0.5, MCF-7/182^R-6, MCF-7/TAM^R-1	Cell cycle
CDC25C-	*cell division cycle 25 homolog C (S.*	MCF-7/S0.5, MCF-7/182^R-6	Cell cycle

Supplementary Table-1

	pombe)		
CDC7-	*cell division cycle 7 homolog (S. cerevisiae)*	MCF-7/S0.5, MCF-7/182^R-6	Cell cycle
CCNA2-	*cyclin A2*	MCF-7/S0.5, MCF-7/182^R-6, MCF-7/TAM^R-1	Cell cycle
CCNB1-	*cyclin B1*	MCF-7/S0.5, MCF-7/182^R-6, MCF-7/TAM^R-1	Cell cycle, p53
CCNB2-	*cyclin B2*	MCF-7/S0.5, MCF-7/182^R-6, MCF-7/TAM^R-1	Cell cycle, p53
CDK2-	*cyclin-dependent kinase 2*	MCF-7/S0.5, MCF-7/182^R-6	Cell cycle, p53
ESPL1-	*extra spindle pole bodies homolog 1 (S. cerevisiae)*	MCF-7/S0.5, MCF-7/182^R-6, MCF-7/TAM^R-1	Cell cycle
ORC3L-	*origin recognition complex, subunit 3-like (yeast)*	MCF-7/S0.5	Cell cycle
PTTG1-	*pituitary tumor-transforming 1; pituitary tumor-transforming 2*	MCF-7/S0.5, MCF-7/182^R-6, MCF-7/TAM^R-1	Cell cycle
PKMYT1-	*protein kinase, membrane associated tyrosine/threonine 1*	MCF-7/S0.5, MCF-7/182^R-6	Cell cycle

Supplementary Table-1

DDB2+	*damage-specific DNA binding protein 2, 48kDa*	MCF-7/S0.5, MCF-7/182^{R}-6, MCF-7/TAM^{R}-1	NER, P53
XPC+	*xeroderma pigmentosum, complementation group C*	MCF-7/S0.5, MCF-7/182^{R}-6, MCF-7/TAM^{R}-1	NER
HMGB1L1	*high-mobility group box 1-like 1*	MCF-7/S0.5, MCF-7/182^{R}-6	BER
PARP2-	*poly (ADP-ribose) polymerase 2*	MCF-7/S0.5, MCF-7/182^{R}-6	BER
UNG-	*uracil-DNA glycosylase*	MCF-7/S0.5, MCF-7/182^{R}-6	BER
MSH6-	*mutS homolog 6 (E. coli)*	MCF-7/S0.5, MCF-7/182^{R}-6	MMR
BLM-	*Bloom syndrome, RecQ helicase-like*	MCF-7/S0.5, MCF-7/182^{R}-6	HR
RAD51C-	*RAD51 homolog C (S. cerevisiae)*	MCF-7/S0.5	HR
RAD54L-	*RAD54-like (S. cerevisiae)*	MCF-7/S0.5, MCF-7/182^{R}-6	HR
XRCC3-	*X-ray repair complementing defective repair in Chinese hamster cells 3*	MCF-7/S0.5	HR
DUT-	*deoxyuridine triphosphatase*	MCF-7/S0.5, MCF-7/182^{R}-6, MCF-7/TAM^{R}-1	Pyrimidine metabolism
RRM1-	*ribonucleotide reductase M1*	MCF-7/S0.5	Pyrimidine metabolism, Purine metabolism

Supplementary Table-1

RRM2B+	*ribonucleotide reductase M2 B (TP53 inducible)*	MCF-7/S0.5, MCF-7/182^R-6	Pyrimidine metabolism, P53
RRM2-	*ribonucleotide reductase M2 polypeptide*	MCF-7/S0.5, MCF-7/182^R-6, MCF-7/TAM^R-1	Pyrimidine metabolism, Purine metabolism, P53
TK1-	*thymidine kinase 1, soluble*	MCF-7/S0.5, MCF-7/182^R-6, MCF-7/TAM^R-1	Pyrimidine metabolism
TYMS-	*thymidylate synthetase*	MCF-7/S0.5	Pyrimidine metabolism
PRPS2-	*phosphoribosyl pyrophosphate synthetase 2*	MCF-7/S0.5	Purine metabolism
BAX+	*BCL2-associated X protein*	MCF-7/S0.5, MCF-7/182^R-6, MCF-7/TAM^R-1	P53
SESN1+	*sestrin 1*	MCF-7/S0.5, MCF-7/182^R-6, MCF-7/TAM^R-1	P53
GTSE1-	*G-2 and S-phase expressed 1*	MCF-7/S0.5, MCF-7/182^R-6	P53
TUBA1B-	*hypothetical gene supported by AF081484; NM_006082; tubulin, alpha 1b*	MCF-7/S0.5, MCF-7/182^R-6	Gap junction
TUBA1A-	*tubulin, alpha 1a*	MCF-7/S0.5, MCF-7/182^R-6	Gap junction
TUBA1C-	*tubulin, alpha 1c*	MCF-7/S0.5, MCF-7/182^R-6	Gap junction

Supplementary Table-1

TUBA3D-	*tubulin, alpha 3d; tubulin, alpha 3c*	MCF-7/S0.5, MCF-7/182^{R}-6	Gap junction
TUBB2C-	*tubulin, beta 2C*	MCF-7/S0.5, MCF-7/182^{R}-6	Gap junction
TUBB6-	*tubulin, beta 6*	MCF-7/S0.5	Gap junction
TUBB4Q-	*tubulin, beta polypeptide 4, member Q*	MCF-7/S0.5, MCF-7/182^{R}-6	Gap junction
TUBB-	*tubulin, beta; similar to tubulin, beta 5; tubulin, beta pseudogene 2; tubulin, beta pseudogene 1*	MCF-7/S0.5, MCF-7/182^{R}-6	Gap junction
RPA2-	*replication protein A2, 32kDa*	MCF-7/182^{R}-6	DNA replication, NER, MMR, HR
GADD45A+	*growth arrest and DNA-damage-inducible, alpha*	MCF-7/182^{R}-6, MCF-7/TAM^{R}-1	Cell cycle, P53
SMC3-	*structural maintenance of chromosomes 3*	MCF-7/182^{R}-6	Cell cycle
CCNG1+	*cyclin G1*	MCF-7/182^{R}-6	P53
CCNG2+	*cyclin G2*	MCF-7/182^{R}-6	P53
LSM4-	*LSM4 homolog, U6 small nuclear RNA associated (S. cerevisiae)*	MCF-7/182^{R}-6	Spliceosome
PRPF3-	*PRP3 pre-mRNA processing factor 3 homolog (S. cerevisiae)*	MCF-7/182^{R}-6	Spliceosome

Supplementary Table-1

THOC4-	*THO complex 4*	MCF-7/182^R-6	Spliceosome
PPIH-	*peptidylprolyl isomerase H (cyclophilin H)*	MCF-7/182^R-6	Spliceosome
SFRS1-	*splicing factor, arginine/serine-rich 1*	MCF-7/182^R-6	Spliceosome
SFRS2-	*splicing factor, arginine/serine-rich 2*	MCF-7/182^R-6	Spliceosome
SFRS4-	*splicing factor, arginine/serine-rich 4*	MCF-7/182^R-6	Spliceosome
TP53I3+	*tumor protein p53 inducible protein 3*	MCF-7/TAM^R-1	P53
FMO5+	*flavin containing monooxygenase 5*	MCF-7/TAM^R-1	Drug metabolism
GSTK1+	*glutathione S-transferase kappa 1*	MCF-7/TAM^R-1	Drug metabolism
MAOA+	*monoamine oxidase A*	MCF-7/TAM^R-1	Drug metabolism
MAD2L1-	*MAD2 mitotic arrest deficient-like 1 (yeast)*	MCF-7/S0.5, MCF-7/182R-6, MCF-7/TAI	Cell cycle
RAD21-	*RAD21 homolog (S. pombe)*	MCF-7/S0.5, MCF-7/182R-6	Cell cycle
SKP2-	*S-phase kinase-associated protein 2 (p45)*	MCF-7/S0.5	Cell cycle
TTK-	*TTK protein kinase*	MCF-7/S0.5, MCF-7/182R-6, MCF-7/TAI	Cell cycle

Supplementary Table-1

BUB1-	*budding uninhibited by benzimidazoles 1 homolog (yeast)*	MCF-7/S0.5, MCF-7/182R-6, MCF-7/TAI	Cell cycle
BUB1B-	*budding uninhibited by benzimidazoles 1 homolog beta (yeast)*	MCF-7/S0.5, MCF-7/182R-6, MCF-7/TAI	Cell cycle
CDC20-	*cell division cycle 20 homolog (S. cerevisiae)*	MCF-7/S0.5, MCF-7/182R-6, MCF-7/TAI	Cell cycle
CDC25C-	*cell division cycle 25 homolog C (S. pombe)*	MCF-7/S0.5, MCF-7/182R-6	Cell cycle
CDC7-	*cell division cycle 7 homolog (S. cerevisiae)*	MCF-7/S0.5, MCF-7/182R-6	Cell cycle
CCNA2-	*cyclin A2*	MCF-7/S0.5, MCF-7/182R-6, MCF-7/TAI	Cell cycle
CCNB1-	*cyclin B1*	MCF-7/S0.5, MCF-7/182R-6, MCF-7/TAI	Cell cycle, p53
CCNB2-	*cyclin B2*	MCF-7/S0.5, MCF-7/182R-6, MCF-7/TAI	Cell cycle, p53
CDK2-	*cyclin-dependent kinase 2*	MCF-7/S0.5, MCF-7/182R-6	Cell cycle, p53
ESPL1-	*extra spindle pole bodies homolog 1 (S. cerevisiae)*	MCF-7/S0.5, MCF-7/182R-6, MCF-7/TAI	Cell cycle
ORC3L-	*origin recognition complex, subunit 3-like (yeast)*	MCF-7/S0.5	Cell cycle

Supplementary Table-1

PTTG1-	*pituitary tumor-transforming 1; pituitary tumor-transforming 2*	MCF-7/S0.5, MCF-7/182R-6, MCF-7/TAI	Cell cycle
PKMYT1-	*protein kinase, membrane associated tyrosine/threonine 1*	MCF-7/S0.5, MCF-7/182R-6	Cell cycle
DDB2+	*damage-specific DNA binding protein 2, 48kDa*	MCF-7/S0.5, MCF-7/182R-6, MCF-7/TAI	NER, P53
XPC+	*xeroderma pigmentosum, complementation group C*	MCF-7/S0.5, MCF-7/182R-6, MCF-7/TAI	NER
HMGB1L1	*high-mobility group box 1-like 1*	MCF-7/S0.5, MCF-7/182R-6	BER
PARP2-	*poly (ADP-ribose) polymerase 2*	MCF-7/S0.5, MCF-7/182R-6	BER
UNG-	*uracil-DNA glycosylase*	MCF-7/S0.5, MCF-7/182R-6	BER
MSH6-	*mutS homolog 6 (E. coli)*	MCF-7/S0.5, MCF-7/182R-6	MMR
BLM-	*Bloom syndrome, RecQ helicase-like*	MCF-7/S0.5, MCF-7/182R-6	HR
RAD51C-	*RAD51 homolog C (S. cerevisiae)*	MCF-7/S0.5	HR
RAD54L-	*RAD54-like (S. cerevisiae)*	MCF-7/S0.5, MCF-7/182R-6	HR
XRCC3-	*X-ray repair complementing defective repair in Chinese hamster cells 3*	MCF-7/S0.5	HR

Supplementary Table-1

DUT-	*deoxyuridine triphosphatase*	MCF-7/S0.5, MCF-7/182R-6, MCF-7/TAI	Pyrimidine metabolism
RRM1-	*ribonucleotide reductase M1*	MCF-7/S0.5	Pyrimidine metabolism, Purine metabolism
RRM2B+	*ribonucleotide reductase M2 B (TP53 inducible)*	MCF-7/S0.5, MCF-7/182R-6	Pyrimidine metabolism, P53
RRM2-	*ribonucleotide reductase M2 polypeptide*	MCF-7/S0.5, MCF-7/182R-6, MCF-7/TAI	Pyrimidine metabolism, Purine metabolism, P5
TK1-	*thymidine kinase 1, soluble*	MCF-7/S0.5, MCF-7/182R-6, MCF-7/TAI	Pyrimidine metabolism
TYMS-	*thymidylate synthetase*	MCF-7/S0.5	Pyrimidine metabolism
PRPS2-	*phosphoribosyl pyrophosphate synthetase 2*	MCF-7/S0.5	Purine metabolism
BAX+	*BCL2-associated X protein*	MCF-7/S0.5, MCF-7/182R-6, MCF-7/TAI	P53
SESN1+	*sestrin 1*	MCF-7/S0.5, MCF-7/182R-6, MCF-7/TAI	P53
GTSE1-	*G-2 and S-phase expressed 1*	MCF-7/S0.5, MCF-7/182R-6	P53
TUBA1B-	*hypothetical gene supported by AF081484; NM_006082; tubulin, alpha 1b*	MCF-7/S0.5, MCF-7/182R-6	Gap junction
TUBA1A-	*tubulin, alpha 1a*	MCF-7/S0.5, MCF-7/182R-6	Gap junction

Supplementary Table-1

TUBA1C-	*tubulin, alpha 1c*	MCF-7/S0.5, MCF-7/182R-6	Gap junction
TUBA3D-	*tubulin, alpha 3d; tubulin, alpha 3c*	MCF-7/S0.5, MCF-7/182R-6	Gap junction
TUBB2C-	*tubulin, beta 2C*	MCF-7/S0.5, MCF-7/182R-6	Gap junction
TUBB6-	*tubulin, beta 6*	MCF-7/S0.5	Gap junction
TUBB4Q-	*tubulin, beta polypeptide 4, member Q*	MCF-7/S0.5, MCF-7/182R-6	Gap junction
TUBB-	*tubulin, beta; similar to tubulin, beta 5; tubulin, beta pseudogene 2; tubulin, beta pseudogene 1*	MCF-7/S0.5, MCF-7/182R-6	Gap junction
RPA2-	*replication protein A2, 32kDa*	MCF-7/182R-6	DNA replication, NER, MMR, HR
GADD45A+	*growth arrest and DNA-damage-inducible, alpha*	MCF-7/182R-6, MCF-7/TAMR-1	Cell cycle, P53
SMC3-	*structural maintenance of chromosomes 3*	MCF-7/182R-6	Cell cycle
CCNG1+	*cyclin G1*	MCF-7/182R-6	P53
CCNG2+	*cyclin G2*	MCF-7/182R-6	P53
LSM4-	*LSM4 homolog, U6 small nuclear RNA associated (S. cerevisiae)*	MCF-7/182R-6	Spliceosome

Supplementary Table-1

PRPF3-	*PRP3 pre-mRNA processing factor 3 homolog (S. cerevisiae)*	MCF-7/182R-6	Spliceosome
THOC4-	*THO complex 4*	MCF-7/ICI	Spliceosome
PPIH-	*peptidylprolyl isomerase H (cyclophilin H)*	MCF-7/182R-6	Spliceosome
SFRS1-	*splicing factor, arginine/serine-rich 1*	MCF-7/182R-6	Spliceosome
SFRS2-	*splicing factor, arginine/serine-rich 2*	MCF-7/182R-6	Spliceosome
SFRS4-	*splicing factor, arginine/serine-rich 4*	MCF-7/182R-6	Spliceosome
TP53I3+	*tumor protein p53 inducible protein 3*	MCF-7/TAMR-1	P53
FMO5+	*flavin containing monooxygenase 5*	MCF-7/TAMR-1	Drug metabolism
GSTK1+	*glutathione S-transferase kappa 1*	MCF-7/TAMR-1	Drug metabolism
MAOA+	*monoamine oxidase A*	MCF-7/TAMR-1	Drug metabolism

Supplementary Table 1: The list of differentially expressed genes involved in critical biological pathways in MCF-7/S0.5, MCF-7/TAM^{R}-1 and MCF-7/182^{R}-6 cells. "+" – the gene is up-regulated; "-" – the gene is down-regulated.
